# Omnidirectional soft pneumatic actuators: a design and optimization framework

**DOI:** 10.3389/frobt.2024.1418484

**Published:** 2024-09-10

**Authors:** Maria Moutousi, Panagiotis Polygerinos

**Affiliations:** Control Systems and Robotics Laboratory (CSRL), Department of Mechanical Engineering, School of Engineering, Hellenic Mediterranean University, Heraklion, Greece

**Keywords:** design framework, design optimization, response surface methodology, finite element analysis, central composite design, soft pneumatic actuators, omnidirectional soft actuators

## Abstract

**Introduction:**

Soft pneumatic actuators (SPAs) play a pivotal role in soft robotics due to their unique characteristics of compliance, flexibility, and adaptability. There are plenty of approaches that examine the modeling parameters of SPAs, aiming to optimize their design and, thus, achieve the most advantageous responses. Current optimization methods applied to SPAs are usually performed individually for each design parameter without considering the simultaneous effect all parameters can have on the output performance. This modeling shortcoming is essential to be addressed since customized SPAs are used in a variety of applications, each with different output requirements.

**Methods:**

This study provides a generalized design optimization framework for modeling the SPA performance for their motion profiles, the produced strain energy while being deformed, and their stiffness characteristics. Utilizing experimentally validated finite element methods, all geometrical and material parameters of the models are investigated in response surface methodology optimization using the central composite design approach.

**Results:**

The results showcase the entire design space of omnidirectional SPAs, along with their output performance, providing guidelines to the end user for design optimization.

**Discussion:**

The offering of this modeling process for SPAs can be adapted to the demands of any potential application and ensure the best performance with respect to the required output responses.

## 1 Introduction

For the past couple of decades, soft robotic technology has been oriented toward setting up robotic mechanisms made of highly deformable materials. These mechanisms are characterized by high dexterity and safety, exhibit a variety of motions that offer applicability to a wide range of tasks including dynamic tasks, and are sensitive to the physical interaction, allowing usage in difficult-to-access environments. The effectiveness of these soft robotic mechanisms hinges on the performance of their actuators. To date, soft pneumatic actuators (SPAs) are the most common and widely used soft actuators. SPAs have internal cavities, i.e., chambers, for pneumatic actuation, which can be deformed using negative or positive pressures. The pneumatic actuation approach is particularly favored due to its numerous advantages, including lightweight construction, rapid response times, and straightforward and cost-effective implementation, as well as robust pressure resistance (Xavier et al., 2022). SPAs perform motions such as bending, elongation, contraction, twisting, and combinations of the aforementioned. They can be manufactured by silicone rubber casting approaches, directly by 3D-printing methods using flexible filaments or elastomeric resins, or by thermal bonding of thermoplastics (Niiyama et al., 2015). SPAs find utilization in a range of applications, including minimally invasive surgery (Shi et al., 2022; Decroly et al., 2021; Kwok et al., 2022), rehabilitation (Polygerinos et al., 2013; Cianchetti et al., 2013; Fidinillah et al., 2023), safe human–robot interaction scenarios (Noritsugu, 2005; Ku et al., 2024), biomedical devices (Greef and Delchambre, 2009), and handling of delicate objects (Shintake et al., 2018; Deimel and Brock, 2013; Zhou et al., 2021).

The most popular categories of SPAs are the reinforced and pneumatic network (PneuNet) actuators. A well-known subcategory of SPAs comprises omnidirectional actuators, which were proposed by Suzumori et al. (1991a) and Suzumori et al. (1991b) and continue to attract considerable scholarly attention (Xavier et al., 2021b; Lee et al., 2024; Luo et al., 2023). In the existing literature, the most common omnidirectional actuators are monolithic structures with multiple internal pneumatic/hydraulic chambers (Abidi et al., 2018; Decroly et al., 2021). However, they can also be formed through assemblies of multiple single SPAs connected in series or parallel (O’Brien and Lane, 2001; Kalisky et al., 2017) or in the form of polyhedral networks (Liu et al., 2024).

The design and the modeling of SPAs used in various applications and how it is feasible to optimize their design to achieve certain responses have been extensively discussed by a great number of researchers. In particular, numerous studies have investigated the effect of design parameters, either geometrical or structural, on responses such as the bending performance and the force output of SPAs (Elsayed et al., 2014b; Polygerinos et al., 2015; Shi et al., 2022). In these studies, the most frequent module geometry is chosen as a cylinder with internal chambers (Elsayed et al., 2014a) acting as the pressurization vessels. Aside from this type of SPAs, PneuNets are also examined extensively (Hu et al., 2018; Zhu et al., 2022; Jing et al., 2022; Fidinillah et al., 2023). In the design optimization stages, this is implemented in most cases by using finite element analysis (Yan et al., 2016; Hu et al., 2018; Fidinillah et al., 2023). In there, each geometrical parameter is examined sequentially with respect to the desired output response by testing a range of values while preserving fixed values for the rest. However, this approach may lead to different output results depending on the sequence of investigation being followed. Therefore, this approach fails to consider all the factors that affect the SPA behavior simultaneously. Additionally, every application carries its unique requirements for SPAs, which need to be satisfied under optimum conditions. In this context, there is a lack of a generalized framework regarding the design methodology that could be easy to customize to meet application-specific requirements and achieve the desired responses as well. In this study, we aim to address this need and take the initial stride toward an overall fundamental design methodology of SPAs for optimizing responses such as bending ability, strain energy consumption, and stiffness, which constitute major key elements for evaluating and characterizing the SPA performance and profiles (Fidinillah et al., 2023; Zhu et al., 2022; Jing et al., 2022), based on each application demand, so far lacking in the scientific literature.

In this work, we contribute to a comprehensive design framework for omnidirectional SPAs, and the paper is structured as follows: Section 2.1 focuses on exploring a wide parameter space associated with the modeling of such actuators and providing broader design, geometrical, and structural recommendations for SPAs that the end user could take under consideration; in Section 2.2, guided by the central composite design approach, we simulate omnidirectional actuators with different ranges of values for their constituent geometrical parameters, as well as shore hardness material properties, by using finite element method (FEM) analysis under fully parameterized models. These responses are evaluated and optimized using a response surface methodology (RSM), after experimentally validating the FEM simulation results in Section 2.3; and in Section 3, we use analysis of variance (ANOVA) to examine their responses regarding bending angle performance, actuating pressure-produced strain energy, and the stiffness variation that can be delivered with respect to the parameters under investigation. In this way, we provide a complete guide to the end user for optimizing the behavior of their soft omnidirectional actuators.

## 2 Materials and methods

### 2.1 SPA concept design–parameter space

**FIGURE 1:**
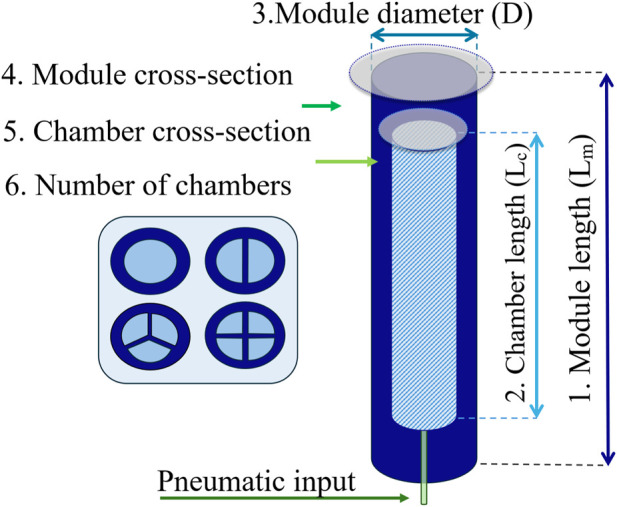
SPA design and geometrical parameter space.

As a starting point, the geometrical parameter space is set up for the desired configuration of the actuator. In this framework study, an exemplary SPA design is adopted, but their parameters can be modified according to specific needs and applications. Here, it is considered as a monolithic module with internal chambers and two distal caps attached to both sides. The module has the following geometrical parameters to consider, as shown in Figure 1: 1) the module length ( $L_{m}$ ); 2) the chamber length ( $L_{c}$ ); 3) the diameter of the module ( D ); 4) the module cross-section shape; 5) the chamber cross-section shape; and 6) the number of chambers.

#### 2.1.1 Fixed parameters

The module length $\left(L_{m}\right)$ , chamber length $\left(L_{c}\right)$ , and external module diameter (D) of the cylindrical omnidirectional soft actuator are restricted to certain values in the centimeter scale so that the experimental validation analysis can be easily facilitated, and the responses are visualized and tracked by a motion capture system with submillimeter accuracy. Furthermore, the chosen values for the module length and chamber length are based, first, on maintaining the ratio $\frac{chamber\;length}{module\;length}$ smaller than 80% so as to achieve a minimum radial expansion (ballooning effect). Excessive ballooning in a SPA consumes energy toward radial expansion rather than contributing to the desired motion or stiffness (Elsayed et al., 2014b). Maintaining this ratio small also reduces the required actuation pressure for achieving the desired SPA configuration (Elsayed et al., 2014b). The existence of solid end caps prevents axial bulging of the material and, therefore, the loss of energy in a non-desirable action.

**FIGURE 2:**
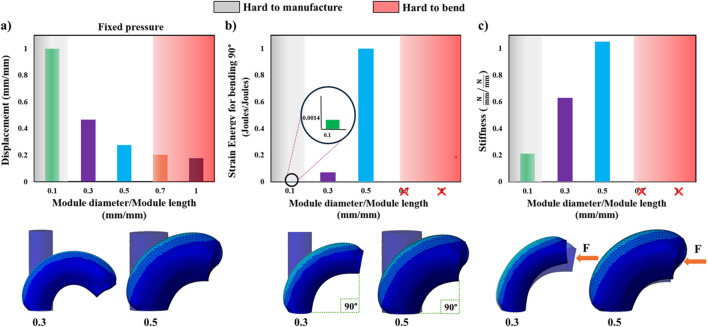
The SPA module diameter-to-module length ratio is tested using FEM simulations for five ratios (0.1, 0.3, 0.5, 0.7, and 1) to investigate **(A)** their bending ability at constant pressure (displacement values closer to 1 indicate a SPA that bends easier), **(B)** their energy consumption profile (strain energy values closer to 1 indicate a SPA that generates more internal strains to bend), and **(C)** their stiffness response when subjected to external pushing force (stiffness values closer to 1 indicate a SPA that deflects less). Ratios of 0.7 and 1 cannot be simulated reaching 90° and, therefore, are omitted in **(B,C)**. The gray and red zones correspond to ranges where there is difficulty for SPAs due to the small dimensions to be fabricated via conventional fabrication methods or bend due to similar dimensions in the two orthogonal axes, respectively. Indicatively, FEM of SPAs with 0.3 and 0.5 ratios is visualized below the graphs for each case.

Additionally, in this study, several $\frac{module\;diameter}{module\;length}$ ratios are tested in various ratio scenarios, using FEM analysis, to provide the end user with a trustworthy overall behavior relation for these parameters. These scenarios refer to bending performance, produced strain energy for reaching a 90° bending angle, and the stiffness profile of the SPA when an external force is applied at its top end-cap surface while pressurized. As shown in Figure 2A, in the scenario where the actuation occurs at a fixed pressure value in one of the chambers of the SPA, the expected displacement increases as the ratio is remains low, i.e., the case of a very long and thin SPA. Figure 2B shows the strain energy produced when pressurizing the SPA at a 90° bending angle, while Figure 2C repeats the previous case but also examines how an external force provokes the deflection of the SPA from the pressurized rested position, i.e., the position at the 90° bending angle. It is observed that a low diameter-to-length ratio facilitates greater bending motion with less produced energy from strain but deflects easier in the case of contact interactions. However, a very small diameter-to-length ratio is not frequently encountered in SPA design studies in the existing literature (Runciman et al., 2019) due to manufacturing difficulties during mold creation and casting or when utilizing other emerging digital fabrication methods (Abidi et al., 2018; Arezzo et al., 2016; Gerboni et al., 2015; Diodato et al., 2018). On the other hand, a higher diameter-to-length ratio produces overall stiffer SPA designs, but it is harder to achieve a wide bending space and consumes greater amounts of energy to do so as well. In this study, the ratio $\frac{module\;diameter}{module\;length}$ is kept at $\frac{1}{5}$ . In particular, the module length is set at 120 mm, chamber length at 100 mm, and module diameter at 25 mm. However, it is worth noting that the above parameters are eventually normalized by dividing with the rest of the relative parameters, and thus, this contributes to the aim of this study, i.e., providing a generalized performance framework that the end user can modify according to their requirements.

#### 2.1.2 Parameters under investigation

**FIGURE 3:**
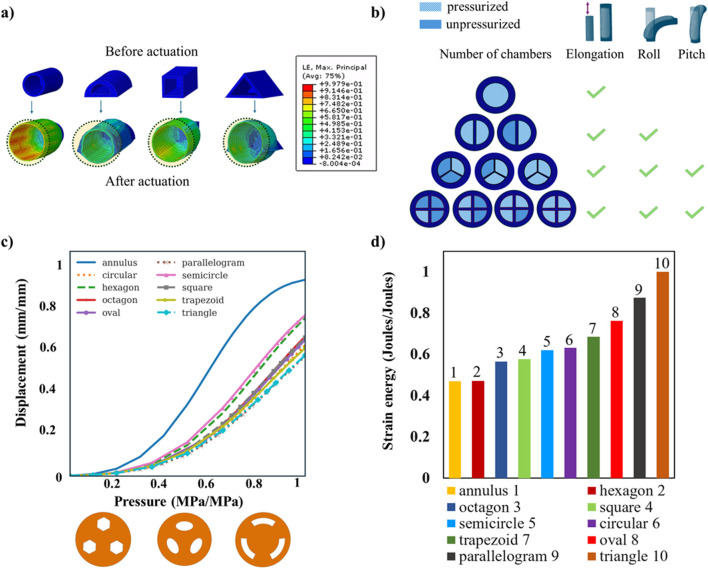
**(**A) FEM analysis of various SPAs with different cross-sectional shapes (circular, semicircular, square, and triangular). When the internal chamber is pressurized, a circular cross-sectional shape is reached in all cases; **(B)** pressurized and unpressurized motion states of SPAs with a different number of chambers. To achieve an omnidirectional SPA design, a minimum of three internal pressurizable chambers are required so that it is steerable in the three-dimensional space offering elongation, roll, and pitch capabilities; **(C)** FEM investigation on various cross sections for omnidirectional SPAs with three chambers (hexagon, oval, and annulus sector cross sections are presented). The cross section of a chamber with an annulus sector design presents the maximum displacement when pressurized. This displacement is associated with the maximum bending ability of the omnidirectional SPA when a single chamber is pressurized; **(D)** FEM investigation as in **(C)** for the amount of strain energy produced when pressurizing a single chamber of the omnidirectional SPA to reach a 90° bending angle. The annulus sector design is shown to generate the least strain in the material that comprises that SPA.

Utilizing FEM analysis, various module cross sections are explored to identify the best module cross section to utilize. All experimented actuator shapes have a single internal concentric chamber, which is identical in shape to their outer shape, and a cross-sectional area equal between all of them. These single-chamber actuators are simulated, assuming hyperelastic properties for their material, and pressure is applied to their cavities. This results in the transformation of all their unpressurized cross sections to a circular cross section when pressurized, as shown in Figure 3A. From an energy perspective, the achieved circular shape geometrically has no edges or corners, so the distribution of stresses and strains is more uniform and does not concentrate in these regions. With only this in mind, any initial cross section can be utilized if one aims to create a single-chamber SPA that, when pressurized, elongates along its main axis. In this instantiation of the SPA, the main criterion for initial geometrical shape selection should therefore be the ease of fabrication each shape could offer, as well as any application-specific limitations. To achieve more than one direction of motion for the SPA, one is required to increase the number of internally pressurizable chambers. As discussed, a single chamber within the actuated SPA makes it elongate along the main axis as the strains are distributed uniformly around the periphery. In the case of two chambers, the SPA can either elongate along its main axis, if both chambers are equally pressurized, or can bend, along a single plane, toward the opposite direction of the pressurized chamber with the highest pressure. The degrees of freedom (DOFs) therefore include elongation and roll. Introducing three or more chambers, the SPA, apart from elongation and roll, gains pitch as a DOF. It is noted that a SPA becomes steerable in the entire three-dimensional space at a minimum of three chambers, but one can opt for additional chambers to maximize the control resolution of the SPA. The correlation between the number of chambers and the DOFs is shown in Figure 3B. Nonetheless, it is significant that aside from these actuation chambers, there is usually the need for an additional chamber that serves the purpose of enabling tools and other components to pass through the entire length of the SPA, such as cameras and grippers in surgical laparoscopic tools (Yan et al., 2016; Cianchetti et al., 2013; Kwok et al., 2022).

For the cross-sectional shape of the chamber(s), the selection can be made based on the following requirements: i) the wall thickness to be of equal size across its span to allow uniform distribution of the stresses toward the outer surface of the module while being pressurized and ii) less material to be used so that the volume of the pressurizable chambers is increased, and thus, the pressure area required to perform bending, or any other combined motion pattern, can be decreased, consequently limiting the strain exerted on the deformed areas of the SPA. Taking these requirements into consideration, for the case of the one-chamber SPA, the optimal chamber cross-section shape derived is circular. In the case of two symmetrical chambers, the best resulting chamber shape is semicircular. Lastly, in the case of three chambers, these are best to be at a 120° symmetrically spaced arrangement with an annulus sector shape. This geometrical distribution of area in the chambers is the logical derivation to occur when one splits the SPA in continuously more chambers while preserving a uniform outer wall thickness. However, in this study, the hypothesis of the annulus sector being the optimal chamber shape for omnidirectional SPAs with three or more chambers is tested against a number of different chamber cross sections of the same area using FEM analysis. Each cross section is tested in two scenarios that, in subsequent sections, are analyzed in detail. The first scenario aims to investigate the maximum allowable bending angle the SPA can reach while only one chamber is pressurized at constant pressure. The second is to examine the energy cost, i.e., the produced strain energy for bending to a 90° angle, when actuating a single chamber. The results of these tests are shown normalized in Figure 3C and Figure 3D, respectively, and highlight the optimal cross section chamber shape within the context of the maximum bending angle and the least produced strain energy, revealing the superiority of the annular sector over all other major shapes. It is also observed that the annulus sector preserves its superiority as a chamber shape for omnidirectional SPAs with a minimum of three pneumatic chambers irrespective of the scale/size of the chamber area. This validation is performed through FEM analysis of the same SPAs by doubling the chamber area, and the results are given in Supplementary Material.

**FIGURE 4:**
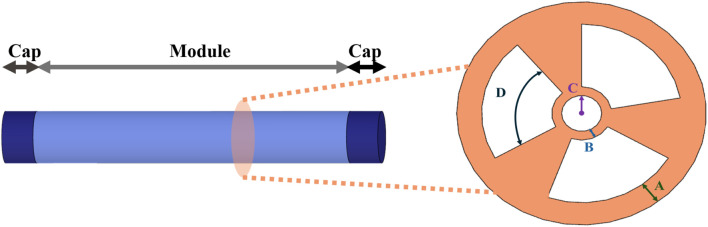
Omnidirectional SPA configuration along with the cross section of the module. Design variables A, B, C, and D represent the wall thickness, inner thickness, central channel radius, and arc angle, respectively.

Design of the selected configuration: The SPA performs best when in a cylindrical form, with at least three pneumatic chambers of an annulus sector cross-sectional shape that are radially arranged around the central axis. Furthermore, the SPA carries one main circular central channel so that various tools can pass through the module and reach the distal end with minimum influence in bending performance, as shown in Figure 4.

#### 2.1.3 Cross-section shape-driving parameters

Adding the additional cavity in the center of the SPA generates the selected cross-section shape with a section of annulus. This leads us to further explore the effect of the driving parameters that define the particular cross section. The outer radius of the annulus sector is associated with the thickness between the outer surface of the module and the outer surface of the chamber of the SPA, defined henceforth as wall thickness. The inner radius of the annulus sector is linked to the thickness between the interior surface of the chamber and the surface of the central channel, defined henceforth as inner thickness. The radius of the central channel is also examined. The final variable to consider is the angle of the arc of the annulus sector. Therefore, a total of four variables are selected to investigate their effect on SPA responses, which are depicted as A,B,C , and D , respectively, in Figure 4.

### 2.2 Response surface methodology for design optimization

The RSM comprises a set of mathematical and statistical tools suitable for modeling and analyzing complex relationships between multiple independent input variables and the responses they produce on the dependent variable Y (Lamidi et al., 2022). The aim is to optimize the response, meaning either minimize unfavorable or undesired responses or maximize the desired responses (Njoku and Otisi, 2023). The RSM belongs to the wider statistical approach of the design of experiments (DoE) for planning, conducting, analyzing, and interpreting data acquired by experiments (Njoku and Otisi, 2023). The planning of an RSM experiment includes the selection of factors (i.e., input variables) that have an impact on the measured responses (i.e., output variables), the selection of factor levels (i.e., the values a factor can take on), determining the range and levels at which each factor will be explored, and the selection of the appropriate experimental design. Such an experiment entails a sequence of tests, referred to as runs, where each input variable is varied at different levels so as to analyze the fluctuations in the output responses. As a result, the RSM provides the advantages of i) using statistical models to approximate the response values for a specific range of independent variables; ii) determining the statistical significance of input variables; and iii) presenting their optimum values that lead to the optimization of the responses (Lamidi et al., 2022). In the RSM, response surfaces are graphical representations used to visualize the interactive effects of process variables and their consequent effects on each response (Ye et al., 2017; Rezaee et al., 2014). There are two major factorial designs in the RSM: the Box–Behnken designs (BBDs) and the central composite designs (CCDs) (Lamidi et al., 2022). Factorial is associated with enabling the investigation of the influence of multiple factors simultaneously, instead of one at a time. Both are used to assess the quadratic response surface and for developing second-order polynomial models in the RSM. The quadratic model is considered the best since it includes linear terms for all factors, squared terms for all factors, and products of all pairs of variables (Njoku and Otisi, 2023) and is described by Equation 1:

$$Y=\beta_{0}+\overset{k}{\underset{i=1}{\sum }}\beta_{i}X_{i}+\overset{k}{\underset{i=1}{\sum }}\beta_{ii}X_{i}^{2}+\overset{k-1}{\underset{i=1}{\sum }}\overset{k}{\underset{j=2}{\sum }}\beta_{ij}X_{i}X_{j}+\epsilon ,$$ (1)

where Y is the output response, k is the number of independent variables, $X_{i}$ is the main effect for factor i , $X_{i}X_{j}$ is their interactions, $X_{i}^{2}$ is their quadratic components, $\beta_{0}$ is the intercept or regression coefficient, $\beta_{i},\beta_{ii}$ and $\beta_{ij}$ are the regression coefficients for the linear, quadratic, and interaction models, respectively, and ϵ is the experimental/residual error.

On the other hand, simple linear and interaction models are sometimes adequate to be considered the approximation relationship between factors (input variables) and responses (output variables) and, therefore, efficiently predict the optimum values. In this study, the process of optimization includes the estimation of coefficients, prediction of responses, and evaluation of the developed model and is performed using the statistical analysis suite Design-Expert 13 (Stat-Ease Inc., Minneapolis, MN, United States).

#### 2.2.1 Central composite design

CCD is used as the experimental design methodology as it facilitates the efficient exploration of the factor space, maintaining a balance between precision and resource requirements. It is a fractional factorial design with a center point attached to a group of stars, or axial points, which allows the estimation of the curvature (Njoku and Otisi, 2023; Bhattacharya, 2021). In particular, four independent/input variables (i.e., factors) are considered, namely, wall thickness (A), inner thickness (B), radius of the central channel (C), and arc angle (D), each with three levels, as shown in Figure 4. The examined *Y* responses of the designated experiment are i) the bending angle at a fixed pressure value; ii) the produced strain energy for the SPA to bend at a 90° bending angle; and iii) the stiffness of the SPA. For the latter response, the aim is to examine the deflection of the SPA, when it has already bent 90°, and then, a force is exerted on its distal top surface. Measuring the displacement of the SPA and given the force applied, one can calculate the stiffness that is equal to the ratio of the force to the provoked deformation. The number of experimental runs (i.e., the series of tests an experiment encompasses) required for CCD is given by $N=2^{k}+2k+nC$ , where N is the number of experiments, k is the number of factors, C represents the central points, and n is the number of times the experiment is repeated (Bhattacharya, 2021). Repetition refers to multiple response measurements taken at the same combination of factor settings during the same experimental run or consecutive runs. Based on four factors ( k=4 ), one central point (C=1) , and one repetition (n=1) , the CCD requires 25 runs (N=25) in total. Each factor comprises three equally spaced levels, and the experimental response data are extracted from standard computationally modeled simulations. In this study, rather than physically constructing 25 SPAs to obtain their responses, we build a FEM model to rapidly iterate through the required runs. Despite the advantages of using such a FEM model, the preparation of the variations in the number of simulations is time-consuming to be performed manually. For this reason, a Python script is composed that automates the generation of FEM models and facilitates the efficient examination of the above-mentioned responses. The Python script is provided in Supplementary Material.

#### 2.2.2 Finite element method analysis

The parametric modeling of the SPA using Python scripting and the conduction of simulations to investigate its behavior in the three aforementioned scenarios are performed using SIMULIA Abaqus FEA. The first case inspects the produced strain energy while the SPA deforms to a bending angle of 90°. In the second case, a force of one unit is applied at the top end-cap surface while the SPA is pressurized at a 90° angle. Thus, it is feasible to measure the deflection that is created and, as a result, extend our study in estimating the stiffness characteristics of the SPA. Lastly, the third case aims to study, given a fixed pressure value, the maximum bending angle the SPA reaches. Consequently, 25 models, which correspond to the number of runs that the CCD generated, are modeled, and their resulting data from the FEM simulations are extracted to complete the corresponding responses in the CCD. Each SPA model is meshed using solid quadratic tetrahedral elements (Abaqus element type C3D10H) with a seed size of 1.5. As for the material, the SPA is a monolithic structure assigned with silicone rubber, which is defined by mass density, hyperelasticity, isotropy, and incompressibility, regarding the requisite material properties. In the examples used to construct the design framework of this study, the Yeoh model is used as the hyperelastic model to capture the highly extensible and elastic behavior of silicone rubber due to its ability to approach large strain problems (Xavier et al., 2021a). The strain energy function of the Yeoh model (U) is expressed in Equation 2:

$$U=\overset{3}{\underset{i=1}{\sum }}C_{i}\cdot {\left(I_{1}-3\right)}^{i}+\overset{3}{\underset{i=1}{\sum }}\frac{1}{D_{i}}{\left(J_{el}-1\right)}^{2i},$$ (2)

where $C_{i},D_{i}$ are material constants, $I_{1}$ is the first invariant of deviatoric strain, and $J_{el}$ is the elastic volume ratio.

### 2.3 Experimental validation of FEM simulations

Physical experiments with the SPAs are performed to validate the accuracy and reliability of the computational models, and their experimental output responses are compared with the corresponding outputs from the FEM simulations. An additional quasistatic investigation is implemented where the values for the A, B, C, and D variables (Figure 4) are preserved to a constant value while testing two different shore hardness hyperelastic materials, namely, Dragon Skin 20A (Smooth-On, Inc.) and Dragon Skin 30A (Smooth-On, Inc.) with elongation at breaks 620% and 364%, respectively. Both have a mass density of $1.08\;*\;10^{-9}Mg/mm^{3}$ . The corresponding Yeoh model coefficients used for Dragon Skin 30A are $C_{1}=0.11$ and $C_{2}=0.007$ and for Dragon Skin 20A, $C_{1}=0.055$ and $C_{2}=0.0015$ . This approach aims to provide additional information to the SPA designer, regarding the SPA behavior that is influenced by the variation in the material properties.

#### 2.3.1 SPA fabrication process

**FIGURE 5:**
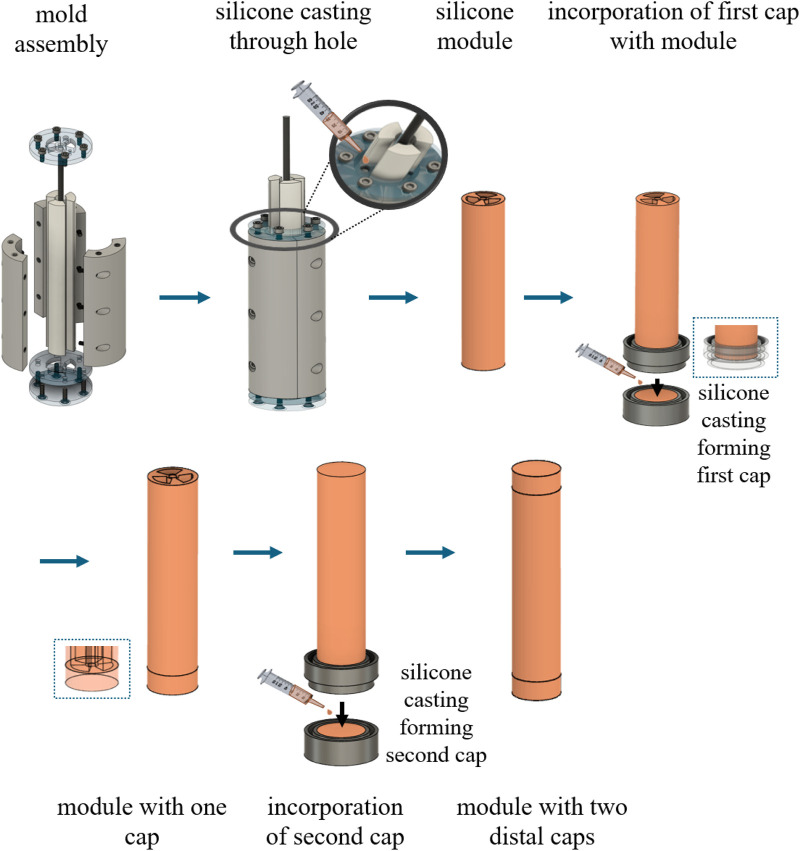
Omnidirectional SPA fabrication process that utilizes multi-part molds for silicone casting.

Two hyperelastic materials, material A (Dragon Skin 30A) and material B (Dragon Skin 20A), are used to fabricate the SPA configuration. Several different SPA fabrication processes are reported in the literature, and for this study, the general approach suggested by Xie et al. (2018); Decroly et al. (2021); Cianchetti et al. (2013); and Yan et al. (2016) is adopted with a few custom modifications. Both silicone elastomer materials require a catalyst to obtain their final solid state. To achieve this, the two parts are mixed together at a 1:1 ratio. The mixture is subjected to vacuum to degas and is then casted in the appropriate additive manufactured mold. Once the silicone is demolded, the SPA module is constructed. To be complete, the module is sealed on both ends with distal end caps. The module is integrated with the distal end cap by placing the module vertically inside a small cylindrical container and filled with silicone at a depth of the desired cap length, as shown in Figure 5. The curing of the silicone is expedited in a convection oven set at 45°C.

#### 2.3.2 FEM simulations vs experiments

**FIGURE 6:**
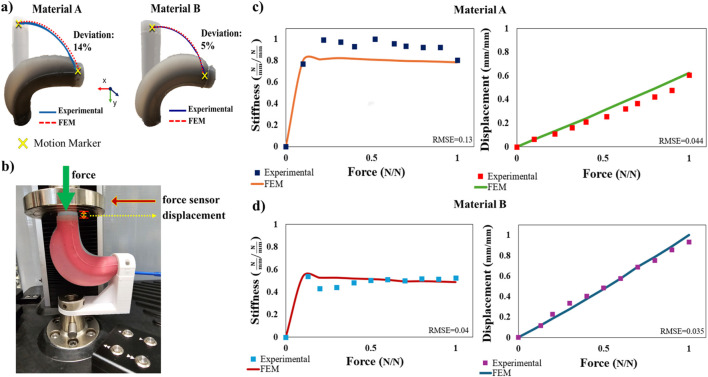
Physical testing of two SPAs with similar geometry but of different shore hardness materials bending at 90°. **(A)** SPA bending trajectories are experimentally obtained using active LED motion markers placed at the distal end and base of the actuators for the ground plane reference. The trajectories are compared with those extracted from FEM simulations presenting a small deviation in both cases. **(B)** Experimental stiffness setup that utilizes a uniaxial force sensor and a linear translation stage. Stiffness values are based on the measurement of force and displacement by the force sensor. **(C,D)** Comparison between experimental and computational results for materials A and B, which illustrate the SPA stiffness and displacement profiles. The values are normalized across the two materials for ease of comparison.

For the bending-angle performance evaluation, a motion capture analysis system (Impulse X2E, PhaseSpace, CA, United States) is used to track the SPA motion in the physical world. Three active motion markers are placed at the base end cap where the SPA is held upright so as to generate a local reference coordinate system. Another single motion marker is placed at the surface of one side of the distal cap of the SPA. The experiment involves a single chamber of the SPA being pressurized until it reaches a 90° bending angle. A comparison between the FEM model and the experimental data is shown in Figure 6A, where the top end-cap trajectories during the particular motions are recorded. The results indicate the good agreement of the FEM model with the physical prototypes for both materials A and B, indicatively, 14% and 5%, respectively. In the SPA case prepared with material A, the deviation concerning the requisite actuation pressure between theoretical and experimental behaviors observed is 9.6%, while in material B, a 11% deviation from the experimental value is spotted in FEM simulation.

For the stiffness investigation, a contact interaction setup, including a universal testing machine, is used to measure the force exerted on the top surface of the SPA and *vice versa*, as shown in Figure 6B. The apparatus contains a digitally fabricated fixture where the SPA rear end cap is secured, while a force sensor can come in contact with the distal end cap. In this way, the force and displacement measurements are recorded by the sensor by gradually compressing the actuated SPA. As shown in Figure 6C, for material A, the root mean square error (RMSE) between the experimental measurements and the FEM simulation results for the stiffness is 13% and 4.4% for the displacement due to force. Similarly, as shown in Figure 6D, for material B, the RMSE is 4% and 3.5% for both stiffness and displacement occurring due to force application, respectively. Comparatively, the displacement of material B, due to its lower shore hardness value, as expected, is greater than the displacement of material A. The stiffness responses present a magnitude-increasing pattern up to a certain force threshold and then starts decreasing. This behavior is observed in both materials A and B, in both FEM simulations and experiments, and can be attributed to the high compliance of the system occurring at the early stages of the contacted force that finds equilibrium as the force increases. Simultaneously, the resistivity at specific local areas of the SPA is also increased due to the compression of its silicone body. As a result, it is feasible to inspect how material properties affect the SPA deflection in the case of contact interactions. However, it needs to be clarified that in the proposed design framework, the optimization process attempts to minimize the displacement due to contact by adjusting all of the investigated parameters while also incorporating material selection as a design parameter according to the desired stiffness profile demands.

## 3 Results

### 3.1 ANOVA and model fit statistics

**FIGURE 7:**
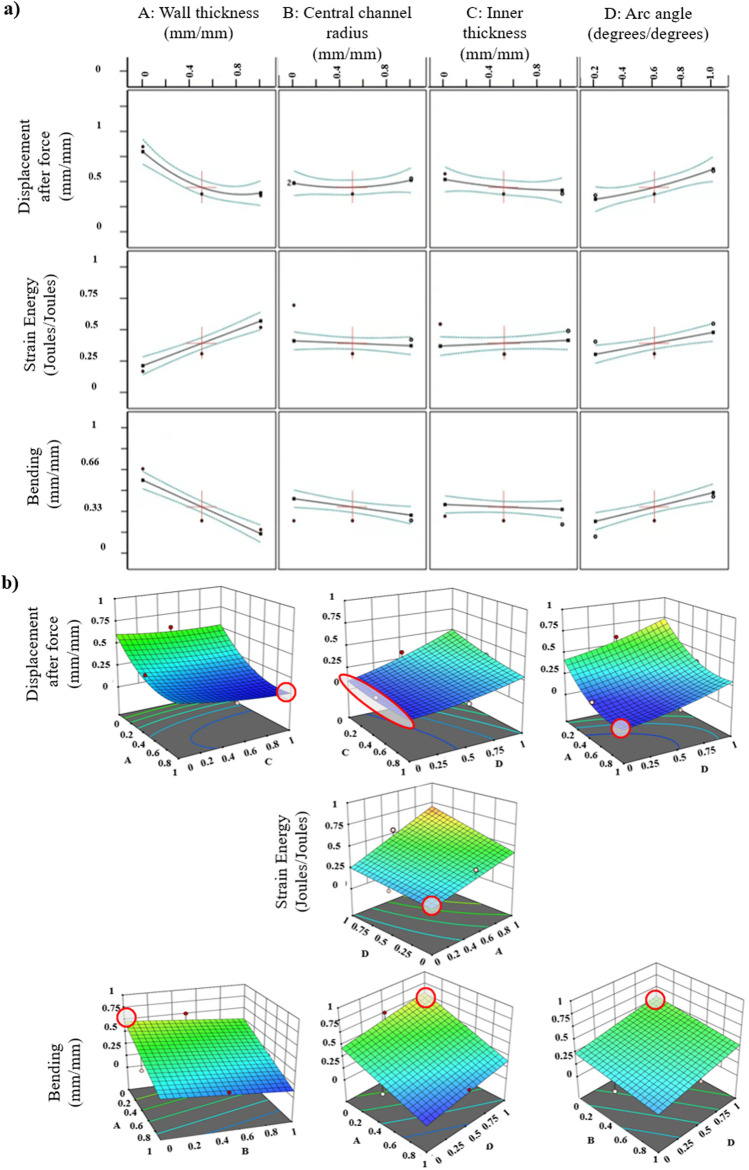
Response surface methodology (RSM) results using a central composite design (CCD) as an optimization approach. **(A)** Correlation of design of experiment (DoE) curves: design variables are presented with respect to each response, i.e., displacement after force applied, strain energy, and bending angle. Axes are normalized between 0 and 1, so 0 indicates the minimum value of the desired range based on the demands of the application an SPA will be used for, and 1 indicates the maximum value correspondingly. **(B)** Interactions of factors (design variables) and their impact on responses. Surface colors represent the gradient range from the lowest, corresponding to blue shades, to the greatest response value, corresponding to red shades. Red dots correspond to response values above the surface, and the pink ones correspond to those below the surface. Red-outlined areas highlight the surface location where the pair of the corresponding variables leads to the optimized, that is, minimization of the displacement of force, minimization of the produced strain energy, and maximization of the bending angle.

The significance of the variables and their interactions is determined using ANOVA with a significance threshold of 95% and a *p*-value of 0.050. The mathematical models are derived using the ANOVA table and used for optimization purposes. In particular, for the response of displacement after the application of force, a quadratic model should be considered initially for model fitting. However, there is the need for some statistical model reduction, indicated by the difference between the predicted R-squared and the adjusted R-squared value being more than 0.2. In the statistical analysis, if the particular difference is less than 0.2, then it is stated that the model can fit the data and can be assuredly used to interpolate. Therefore, the final CCD obtained for this response is a reduced quadratic. For the responses of strain energy and bending angle, the suggested models are linear. The F-value for every model, which is the ratio of the mean square for the individual term to the mean square for the residual, indicates that all the obtained results are statistically significant with also *p*-value variables of less than 0.05 (Saha et al., 2010; Sadhukhan et al., 2016). Moreover, adequate precision is associated with the signal-to-noise ratio (SNR). If the SNR is greater than 4, the model has a strong enough signal to be used for optimization. All the statistical measures used for the examination of the performance of this design framework, i.e., predicted and adjusted R-squared values, model F-values, *p*-values, and adequate precision, are given in a detailed table in Supplementary Material. Apart from that, the model selection along with the statistical significant variables for each response is summarized in Table 1 as they are required to provide a better understanding and justify Figure 7, which is explained in Section 3.2.

**TABLE 1:** Selection of models for each response and their statistical significant terms based on their *p*-value <0.05 .

Response	Displacement after force	Strain energy	Bending angle
Model fit	Reduced quadratic	Linear	Linear
Significant model terms	$A,\;C,\;D,\;CD,\;A^{2}$	A,D	A,B,D

### 3.2 Correlation curves

Figure 7 encapsulates the effect of the investigated independent variables on the aforementioned responses individually and interactively. Analyzing the results shown in Figure 7A, starting from the response of displacement after force, variations in the central channel radius exhibit negligible influence. However, when the wall thickness or inner thickness increases, the displacement decreases. Reversely, when the arc angle increases, the displacement increases as well. As for the response of strain energy, it is observed that as the wall thickness and arc angle increase, the strain energy also increases. The central channel radius and inner thickness do not influence this response significantly. Lastly, concerning the response of bending, when increasing the wall thickness and central channel radius, the bending angle is decreased, while the arc angle, when increasing, leads to its augmentation. The inner thickness, on the other side, does not affect the response. In Figure 7B, two main points are highlighted: 1) the correlations between the independent variables refer only to the statistical significant model terms of the present ANOVA results, depicted in Table 1. The statistical insignificant terms do not influence the responses, so the interaction with them is omitted. This implies that the latter terms may have an impact on SPA behavior; however, with the present DoE, these terms are considered insignificant; 2) for the responses of strain energy and bending angle, where the suggested models are linear, an adjustment from a linear to a two-factor interaction (2FI) model is necessary, including only the products of statistically significant model terms. This adjustment, notably incorporating AD for strain energy and AB,AD,BD for the bending angle, preserves the statistical significance and the accuracy of the model fit, as evidenced by the model fit statistics and ANOVA results shown in Table 1.

## 4 Discussion

In this work, a design methodology and an optimization framework were presented that provide guidelines and insights to the end user working toward the modeling aspects of a SPA. The methodology starts agnostic of cross-sectional shapes and the number of chambers required and provided the geometrical parameter space one must consider according to a specific application in mind for their SPAs. The framework showcased, among many other characteristics, the small importance in the performance of the external shape during pressurization, while the choice of number of pressurizable chambers was found highly correlated with the DOFs one desires to achieve. In the case of those omnidirectional SPAs, which are highly reported in the literature, the minimum number of chambers was determined along with the optimal chamber shape from the perspective of energy and deformation during pressurization. The best performing chamber cross section was found to be the annulus sector. Examining the cross section of an omnidirectional SPA, comprising three pneumatic chambers with an annulus sector shape and a central circular channel that allows application-specific passage through the SPA body, a number of variables were further investigated for their effect on output responses. The variables and their responses focused on the bending performance with a fixed pressure value and the produced strain energy while bending at a fixed 90° angle. Furthermore, the stiffness of the SPA in combination with its deformed shape after pressurization was a topic of interest for many researchers (Shi et al., 2024; Gao et al., 2023; Lamping and de Payrebrune, 2023; Luo et al., 2023), and in this study, the FEM analysis was used to illustrate the stiffness variation across the design spectrum. The investigation used data obtained from the FEM simulations and was implemented using a central composite design, which appertains to the response surface methodology. These design methods enabled us, for the first time, to consider all the variables of the SPA at once, which was underexplored in the existing scientific literature. Yet, in order to consider the FEM approach trustworthy, a series of experimental studies were conducted with physical prototypes, and the performance results were compared to the simulation results, thus validating that the FEM models adequately capture the real-word conditions.

In the experiments, different shore-hardness SPAs were examined to investigate the effect of material properties in these simulation cases. From the existing literature, an important observation was that material model coefficients often vary and cover a wide range (Marechal et al., 2020; Xavier et al., 2021a). Thus, we note that the characterization of hyperelastic materials depends on several factors, such as the fabrication quality of the test specimens, i.e., dog bones, and the conditions of the experiment. In particular, weather conditions, unequal or insufficient mixing of the silicone elastomer with the catalyst, air trapped in the silicone mixture, overcuring, and examination of different ranges of strain and fitted return different material coefficients for the same hyperelastic material models and can lead to these notable variations. In this context, the coefficients in hyperelastic models can occur in two ways. One way is the conventional one, where the coefficients are determined using uniaxial, biaxial, and shear test data. The test data originate from test specimens being deformed (in most cases elongated) by a tensile tester. Therefore, considering all the before-mentioned factors that can affect the coefficient values, the results will satisfy only the specific conditions of the end user’s specific study and application. An alternative option, in case there is no available material testing equipment, one can opt for fine-tuning the existing literature model coefficients, visualizing the SPA behavior via FEM models while obtaining experimental feedback concerning the approximation of the appropriate values.

The ability to optimize the responses according to the requirements of the application should be emphasized while analyzing the impact of these variables on the responses using CCD. Optimization was defined by either maximizing, minimizing, keeping in range, or targeting a value of a response. Regarding the response of the produced strain energy, it is meaningful to minimize first since less strain energy makes the SPA more energy efficient, durable as an overall system, and facilitates more accurate control. One of the advantages of the SPAs is their high compliance. However, the same compliance becomes an unwanted feature once one requires the SPA to interact with the environment. We demonstrated in this study how to passively alter the design parameter/variable to achieve the minimum displacement response when forces act at a SPA. The model provided the desired output parameters for the SPA to preserve high resistance when accepting an external load, something that is desired by many applications at certain stages of SPA utilization. To achieve greater stiffness, the wall thickness and inner thickness of the SPA should be kept close to the upper bound, whereas the arc angle should be kept close to the lower bound. Finally, with respect to the bending motion in free space of the SPA, it is found useful in most cases to maximize the bending angle under a fixed pressure value. Hence, for maximum bending, the wall thickness and central channel radius should be kept low and the arc angle high. Altogether, this optimization process provided an essential component for modern soft robotics, where SPAs and more specifically omnidirectional SPAs find utilization in more and more domains, and thus, the needs and requirements spread across a wide parameter range.

The present study considered exclusively the design, implying that active stiffness control, neither internal nor external, e.g., through reinforcements/constraints, has been applied. Although reinforcements could also be incorporated in this design framework guide, we believe that as a more generalized methodology for SPAs is being developed, it is crucial to deplete all the possible adjustments that a plain hyperelastic material actuator and its geometry can provide in the first place and then proceed to any additional modifications. Future work could seek to include such passive and active reinforcements to further improve the SPA responses and also examine them in dynamic scenarios.

## Data Availability

The original contributions presented in the study are included in the article/Supplementary Material; further inquiries can be directed to the corresponding author.
