# Supplementary material for: Omnidirectional soft pneumatic actuators: a design and optimization framework
Source: Front Robot AI. 2024 Sep 10;11:1418484. doi: 10.3389/frobt.2024.1418484 (PMC11419958; doi:10.3389/frobt.2024.1418484)
Supplement: Supplementary file 1 [file DataSheet1.pdf]

Supplementary Material

1 SUPPLEMENTARY DATA

1.1 Optimal Cross-sectional Chamber Shape

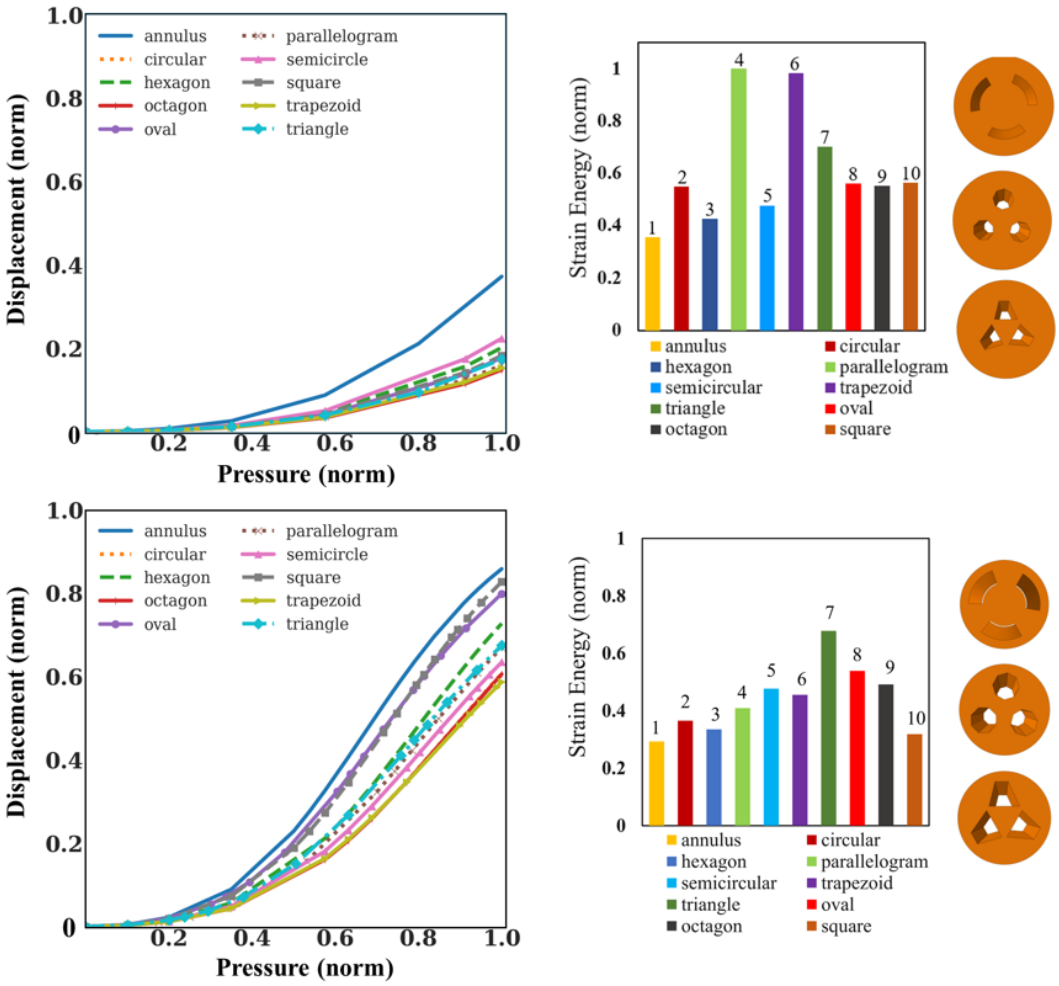

Figure S1. Comparison between different cross-sectional chamber shapes for a chamber with area A and a chamber with double area 2A of the omnidirectional SPA (with 3 chambers as seen from the presented cross-sections). Top row presents the results when actuating a single pneumatic chamber of area A and the bottom row for the same chamber in case of double area 2A. The actuation pressure value is fixed for both cases. In graphs ”Displacement (norm) vs Pressure(norm)”, the displacement is associated with the maximum bending ability of the omnidirectional SPA when a single chamber is pressurized. In graphs ”Strain energy(norm)”, the amount of strain energy produced when pressurizing a single chamber of the omnidirectional SPA to reach a 90° bending angle for each chamber cross-sectional chamber shape. The cross-section of a chamber with an annulus sector design presents the maximum displacement when pressurized and generates the least strain in the material that comprises that SPA in both cases. The values are normalized across the two cases for ease of comparison.. It is important to note, that there is a geometric and practical limit to scaling up the areas of the chambers. When the chambers become sufficiently large and approach the point of overlapping with one another, they occupy a substantial portion of the actuator’s overall cross-section. At this stage, the shape of the chambers becomes inconsequential to the output performance.

1.2 Additional Experimental Validation

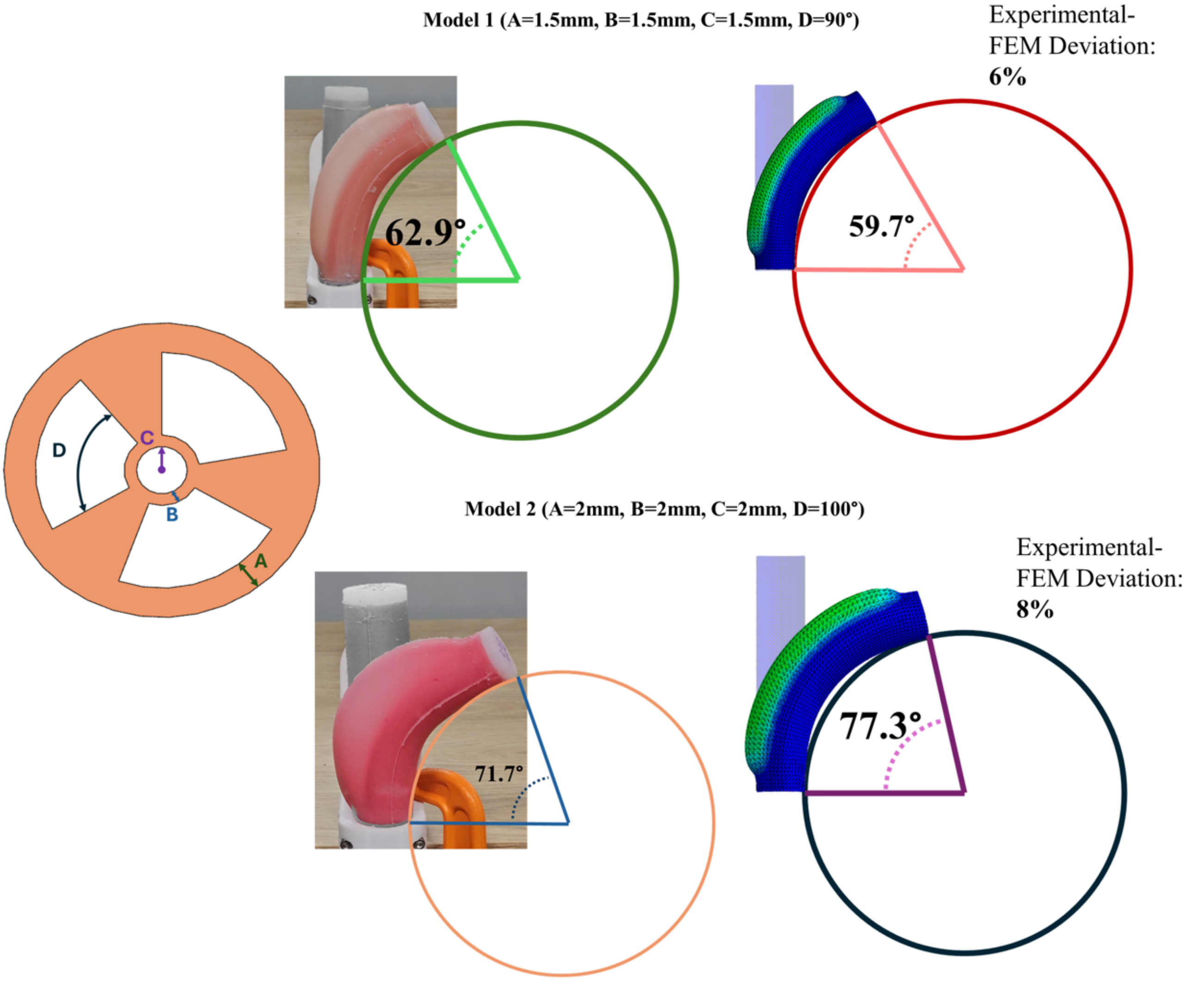

Figure S2. Bending experiment: Comparison of bending angles between experimental and computational results for two different actuator configurations in case of actuating a single chamber of the omnidirectional SPA (with three chambers) with fixed pressure value. The variables under examination can be seen from the SPA's cross-section on the left. It should be noted that based on the RSM optimization methodology, in our work it is stated that increasing A, B, C and D results in a bending angle with an increasing trend. This is validated both experimentally and in FEM.

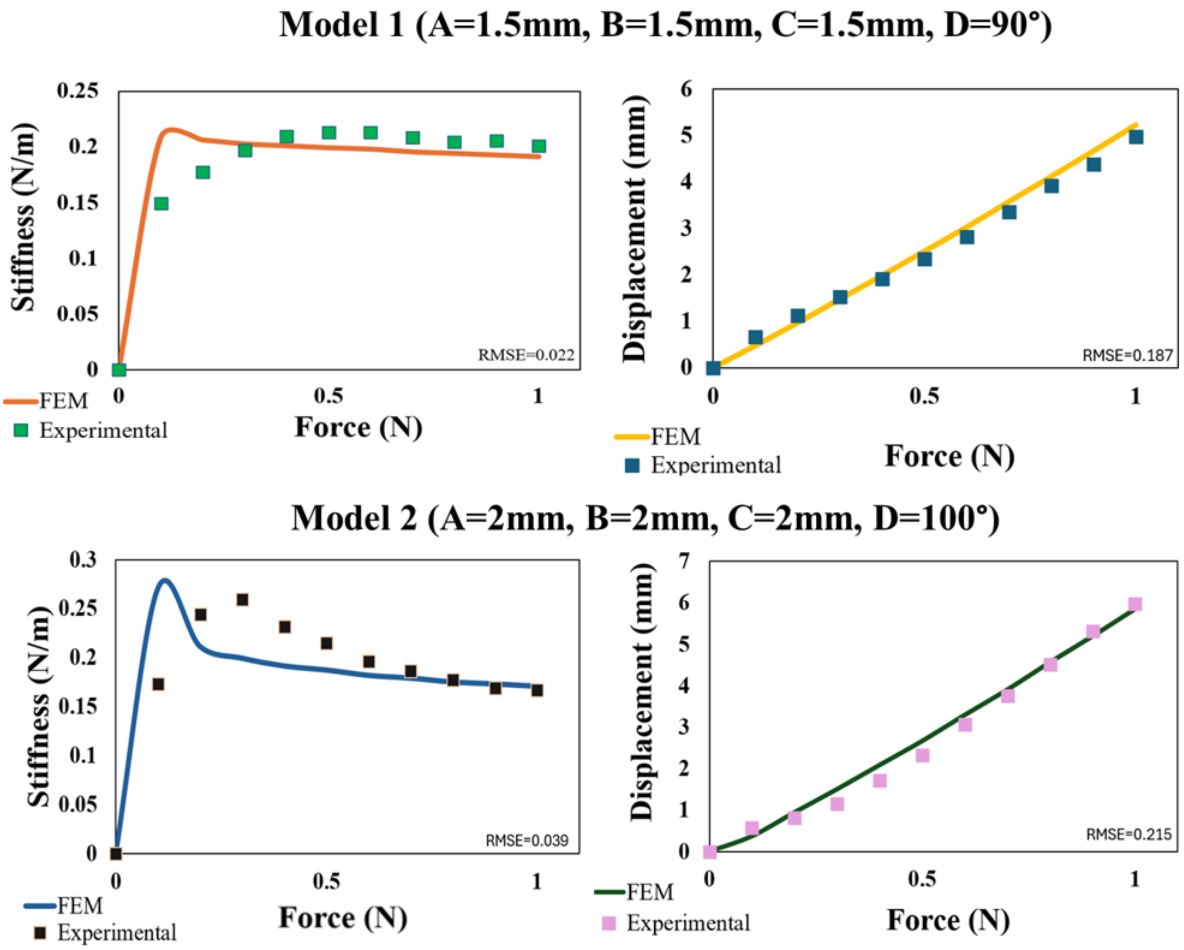

Figure S3. Stiffness investigation: Comparison between experimental and computational results for two different actuator configurations, which illustrate the SPA's stiffness and displacement profiles in case of applying the same force on their top surfaces, while both have already bent 90°. The values are normalized across the two SPA configurations for ease of comparison. RMSE metric for all graphs reveal reliability of the FEM results. Here, based on the RSM optimization methodology, it is stated that increasing A, B results in decreasing the deflection and thus increasing stiffness, while increasing D results in reversed outcome for displacement and stiffness respectively. C variable does not present any major effect . Consequently, it cannot be easily predicted, before validating the case experimentally or computationally, whether Model 1 or 2 presents greater stiffness.

1.3 ANOVA Results

Displacement after force

Model summary statistics

Source	Sequential p-value	Lack of Fit p-value	Adjusted R^2	Predicted R^2	Adeq Precision	
Linear	<0.0001		0.6918	0.6068		
2FI	0.6913		0.6555	0.2366		
Quadratic	0.0015		0.9051	0.7015	14.77	Suggested
Cubic	0.3453		0.9523	-0.3383		Aliased

ANOVA results

Source	Sum of Squares	dif	Mean Square	F-value	p-value	
Model	632.5	13	48.65	20.5	<0.0001	significant
A-wall thickness	305.33	1	305.33	128.67	0.0001	significant
B-central channel	1.69	1	1.69	0.7136	0.4163	
C-inner thickness	20.79	1	20.79	8.76	0.013	significant
D-arc angle	161.66	1	161.66	68.13	0.0001	significant
AB	7.13	1	7.13	3	0.111	
AC	3.01	1	3.01	1.27	0.2844	
AD	1.29	1	1.29	0.5425	0.4768	
BD	7.37	1	7.37	3.1	0.1058	
CD	17.92	1	17.92	7.55	0.019	significant
A^2	22.87	1	22.87	9.64	0.01	significant
B^2	3.25	1	3.25	1.37	0.2664	
C^2	0.6522	1	0.6522	0.2749	0.6105	
D	1.04	1	1.04	0.4375	0.5219	
Residual	26.1	11	2.37			
Cor Total	658.61	24				

Strain energy

Source	Sequential p-value	Lack of Fit p-value	Adjusted R^2	Predicted R^2	Adeq Precision	
Linear	<0.0001		0.6992	0.6307	12.47	Suggested
2FI	0.6316		0.673	0.4878		
Quadratic	0.0919		0.7802	0.5703		
Cubic	0.9004		0.5183	-12.7196		Aliased

Source	Sum of Squares	df	Mean Square	F-value	p-value	
Model	2.91E+06	4	7.27E+05	14.95	< 0.0001	significant
A-wall thickness	2.30E+06	1	2.30E+06	47.17	< 0.0001	significant
B-central channel	27542.04	1	27542.04	0.5661	0.4606	
C-inner thickness	34412.01	1	34412.01	0.7073	0.4103	
D-arc angle	5.52E+05	1	5.52E+05	11.35	0.0031	significant
Residual	9.73E+05	20	48654.64			
Cor Total	3.88E+06	24				

Bending angle

Source	Sequential p-value	Lack of Fit p-value	Adjusted R^2	Predicted R^2	Adeq Precisi on	
Linear	< 0.0001		0.7996	0.7358	17.28	Suggested
2FI	0.6499		0.7802	0.4572		
Quadratic	0.0663		0.8629	0.5533		
Cubic	0.0694		0.9878	0.7157		Aliased

Source	Sum of Squares	df	Mean Square	F-value	p-value	
Model	16441.62	4	4110.41	24.93	<0.0001	significant
A-wall thickness	11813.87	1	11813.87	71.67	0.0001	significant
B-central channel	1121.78	1	1121.78	6.8	0.0168	significant
C-inner thickness	97.14	1	97.14	0.5893	0.4517	
D-arc angle	3408.83	1	3408.83	20.68	0.0002	significant
Residual	3296.96	20	164.85			
Cor Total	19738.58	24				

Figure S4.

Figure S4. The model suggestion derives from the ANOVA. The trustworthiness of the model and the model summary statistics for each response are presented within the blue-bordered boxes, while ANOVA results within the green-bordered boxes. The highlighted segments are the main areas of interest, that guarantee the reliability of the corresponding model.

1.4 Python Parametric Script

```
# ----------------------------------------------------------
# Title: Parametric SPA's Modeling for ABAQUS
# Author: Maria Moutousi
# Affiliation: Hellenic Mediterennean University
# Date: 05/10/2023
# Description: Each combination of factor levels generated by Central
# Composite Design corresponds to a SPA with different cross-section.
# Each SPA model is automatically modeled and tested by applying
# pressure inside one of its three pneumatic chambers.
# Copyright: 2023 Maria Moutousi
# License: MIT License
# ----------------------------------------------------------

from part import *
from material import *
from section import *
from assembly import *
from step import *
from interaction import *
from load import *
from mesh import *
from optimization import *
from job import *
from sketch import *
from visualization import *
from connectorBehavior import *
from odbAccess import *
from array import *
import numbers
import regionToolset
import displayGroupMdbToolset as dgm
import displayGroupOdbToolset as dgo
import xyPlot
import math
import numpy as np
import os
import datetime
import shutil
```

```python
import time
import random
import bisect
import itertools
Mdb()


############# RUNS FROM CCD #############
runs=[]
number_of_runs=25  # insert your number of experimental runs
                   # from corresponding RSM design
for i in range(number_of_runs):
    runs.append(i)

############# DEFINE VARIABLES AND THEIR VALUES #############
wall_thickness=[1,1.6,1,1.6,1,1.6,1,1.6,1,1.6,1,1.6,1,1.6,1,2,1,1.6,1.5,1.5,
↪ #insert your values from RSM design according to range
central_channel=[1,1,2,1.6,1,1,2,1.6,1,1.2,2,1.6,1,1.2,2,2,1.5,1.5,1,2,1.5,1
↪ #insert your values from RSM design according to range
inner_thickness=[1,1,1,1,2,1.6,2,1.6,1,1.2,1,1,2,1.6,2,2,1.5,1.5,1.5,1.5,1,2,
↪ #insert your values from RSM design according to range
arc_angle=[80,80,80,80,80,80,80,80,100,100,100,100,100,100,100,100,90,90,90,
↪ #insert your values from RSM design according to range
module_radius=12.5 #insert your module radius


############# SUPPLEMENTARY VARIABLES #############

inner_chamber=[]
outer_chamber=[]


for (k,l,m,n,o) in zip(wall_thickness,central_channel,
↪  inner_thickness,arc_angle,runs):
    inner_chamber.append(m + l)
    outer_chamber.append(module_radius - k )


############# CREATE MODULE #############
    if (o!=0):
        myModel=mdb.Model(name='Model-%d' %(o+1))
        s=mdb.models['Model-%d'
         ↪  %(o+1)].ConstrainedSketch(name='myNewSketch_%d' %(o+1),
         ↪  sheetSize=200.0)
    else:
```

```
    s =
    ↪  mdb.models['Model-1'].ConstrainedSketch(name='__profile__',
        sheetSize=200.0)
g, v, d, c = s.geometry, s.vertices, s.dimensions, s.constraints
s.setPrimaryObject(option=STANDALONE)
s.CircleByCenterPerimeter(center=(0.0, 0.0), point1=(15.0, 20.0))
s.ObliqueDimension(vertex1=v[0], vertex2=v[1], textPoint=(15.0,
↪   20.0),
    value=12.5)
session.viewports['Viewport: 1'].view.fitView()
s.CircleByCenterPerimeter(center=(0.0, 0.0),
↪   point1=(-1.27965545654297,
    0.595032691955566))
s.ObliqueDimension(vertex1=v[0], vertex2=v[2],
↪   textPoint=(-1.27965545654297,
    0.595032691955566), value=l)
s.ArcByCenterEnds(center=(0.0, 0.0), point1=(0.0, 11.25),
↪   point2=(-11.25,
    -2.5), direction=COUNTERCLOCKWISE)
s.Line(point1=(-10.9821044270695, -2.44046765045988),
↪   point2=(-5.0,
    -1.1111111111112))
s.PerpendicularConstraint(entity1=g[4], entity2=g[5],
↪   addUndoState=False)
s.CircleByCenterPerimeter(center=(0.0, 0.0), point1=(-5.0,
↪  -1.1111111111112))
s.undo()
s.ArcByCenterEnds(center=(0.0, 0.0), point1=(-5.0,
↪  -1.1111111111112), point2=(
    0.0, 5.0), direction=CLOCKWISE)
s.Line(point1=(0.0, 5.12196914294051), point2=(0.0, 11.25))
s.VerticalConstraint(entity=g[7], addUndoState=False)
s.PerpendicularConstraint(entity1=g[6], entity2=g[7],
↪   addUndoState=False)
s.AngularDimension(line1=g[5], line2=g[7],
↪   textPoint=(-1.32378196716309,
    7.25058746337891), value=n)
s.ObliqueDimension(vertex1=v[5], vertex2=v[0], textPoint=(0.0,
↪   0.0),
    value=inner_chamber[o])
s.ObliqueDimension(vertex1=v[3], vertex2=v[0], textPoint=(0.0,
↪   0.0),
    value=outer_chamber[o])
s.radialPattern(geomList=(g[4], g[5], g[6], g[7]), vertexList=(),
↪   number=3,
```

```
        totalAngle=360.0, centerPoint=(0.0, 0.0))

    p = mdb.models['Model-%d' %(o+1)].Part(name='Body',
    ↪   dimensionality=THREE_D,
        type=DEFORMABLE_BODY)
    p = mdb.models['Model-%d' %(o+1)].parts['Body']
    p.BaseSolidExtrude(sketch=s, depth=100.0)  #insert your module's
    ↪   length
    s.unsetPrimaryObject()
    p = mdb.models['Model-%d' %(o+1)].parts['Body']
    session.viewports['Viewport: 1'].setValues(displayedObject=p)


############# CREATE MATERIAL PROPERTIES############
    mdb.models['Model-%d' %(o+1)].Material(name='silicone')
    mdb.models['Model-%d'
    ↪   %(o+1)].materials['silicone'].Density(table=((1.08e-09, ), ))
    ↪   #insert your material's mass density in Mg/(mm)^3
    mdb.models['Model-%d' %(o+1)].materials['silicone'].Hyperelastic(
    ↪   #insert your material coefficients
        materialType=ISOTROPIC, testData=OFF, type=YEOH,
        volumetricResponse=VOLUMETRIC_DATA, table=((0.096,0.0095,0.0,
        ↪   0.0,
        0.0), ))


############# CREATE 1ST CAP  #############
    if (o!=0):
        s=mdb.models['Model-%d'
        ↪   %(o+1)].ConstrainedSketch(name='myNewSketch_%d' %(o+1),
        ↪   sheetSize=200.0)
    else:
        s =
        ↪   mdb.models['Model-1'].ConstrainedSketch(name='__profile__',
            sheetSize=200.0)
    g, v, d, c = s.geometry, s.vertices, s.dimensions, s.constraints
    s.setPrimaryObject(option=STANDALONE)
    s.CircleByCenterPerimeter(center=(0.0, 0.0), point1=(13.75, 12.5))
    s.ObliqueDimension(vertex1=v[0], vertex2=v[1],
    ↪   textPoint=(7.51244354248047,
        7.44800186157227), value=12.5)
    p = mdb.models['Model-%d' %(o+1)].Part(name='cap-1',
    ↪   dimensionality=THREE_D,
        type=DEFORMABLE_BODY)
    p = mdb.models['Model-%d' %(o+1)].parts['cap-1']
```

```
    p.BaseSolidExtrude(sketch=s, depth=10.0) #insert your cap's length
    s.unsetPrimaryObject()
    p = mdb.models['Model-%d' %(o+1)].parts['cap-1']


############# CREATE 2ND CAP #############
    if (o!=0):
        s1=mdb.models['Model-%d'
        ↪ %(o+1)].ConstrainedSketch(name='myNewSketch_%d' %(o+1),
        ↪ sheetSize=200.0)
    else:
        s1 =
        ↪ mdb.models['Model-1'].ConstrainedSketch(name='__profile__',
          sheetSize=200.0)
    g, v, d, c = s1.geometry, s1.vertices, s1.dimensions,
    ↪ s1.constraints
    s1.setPrimaryObject(option=STANDALONE)
    s1.CircleByCenterPerimeter(center=(0.0, 0.0), point1=(18.75,
    ↪ 16.25))
    s1.ObliqueDimension(vertex1=v[0], vertex2=v[1], textPoint=(18.75,
    ↪ 16.25),
        value=12.5)
    p = mdb.models['Model-%d' %(o+1)].Part(name='cap-2',
    ↪ dimensionality=THREE_D,
        type=DEFORMABLE_BODY)
    p = mdb.models['Model-%d' %(o+1)].parts['cap-2']
    p.BaseSolidExtrude(sketch=s1, depth=10.0) #insert your cap's
    ↪ length
    s1.unsetPrimaryObject()
    p = mdb.models['Model-%d' %(o+1)].parts['cap-2']
    a1 = mdb.models['Model-%d' %(o+1)].rootAssembly


   ############# CREATE ASSEMBLY WITH MODULE,1ST & 2ND CAP
   ↪ #############
    p = mdb.models['Model-%d' %(o+1)].parts['Body']
    a1.Instance(name='Body-1', part=p, dependent=ON)
    p = mdb.models['Model-%d' %(o+1)].parts['cap-1']
    a1.Instance(name='cap-1-1', part=p, dependent=ON)
    p = mdb.models['Model-%d' %(o+1)].parts['cap-2']
    a1.Instance(name='cap-2-1', part=p, dependent=ON)
    p1 = a1.instances['cap-1-1']
    p1.translate(vector=(27.5, 0.0, 0.0))
    p1 = a1.instances['cap-2-1']
    p1.translate(vector=(55.0, 0.0, 0.0))
```

```
session.viewports['Viewport: 1'].view.fitView()
session.viewports['Viewport: 1'].view.setValues(nearPlane=190.296,
    farPlane=331.624, cameraPosition=(185.494, 198.034, -12.6121),
    cameraUpVector=(-0.909552, 0.165933, -0.381027),
    ↪  cameraTarget=(27.5,
    -5.72205e-006, 50))
session.viewports['Viewport: 1'].view.setValues(nearPlane=182.37,
    farPlane=339.549, cameraPosition=(175.805, 180.643, 166.076),
    cameraUpVector=(-0.751907, 0.447095, -0.484502),
    ↪  cameraTarget=(27.5,
    -3.8147e-006, 50))
session.viewports['Viewport: 1'].view.setValues(nearPlane=181.546,
    farPlane=340.373, cameraPosition=(54.9451, 111.446, -184.364),
    cameraUpVector=(-0.956959, -0.255542, 0.137577),
    ↪  cameraTarget=(27.5,
    1.52588e-005, 50))
session.viewports['Viewport: 1'].view.setValues(nearPlane=192.102,
    farPlane=329.817, width=96.8631, height=55.1966,
    ↪  cameraPosition=(
    41.3121, 103.248, -189.859), cameraTarget=(13.867, -8.19834,
    ↪  44.505))
a1 = mdb.models['Model-%d' %(o+1)].rootAssembly
a1.translate(instanceList=('cap-1-1', ), vector=(-27.5, 0.0,
↪  -10.0))
session.viewports['Viewport: 1'].view.setValues(nearPlane=180.963,
    farPlane=340.507, width=173.945, height=99.121,
    ↪  cameraPosition=(
    34.0199, 92.6423, -195.756), cameraTarget=(6.57482, -18.8037,
    ↪  38.6079))
session.viewports['Viewport: 1'].view.setValues(nearPlane=172.157,
    farPlane=306.93, cameraPosition=(0.663876, 183.601, 202.78),
    cameraUpVector=(-0.693639, 0.126252, -0.709172),
    ↪  cameraTarget=(6.60354,
    -18.8821, 38.2647))
a1 = mdb.models['Model-%d' %(o+1)].rootAssembly
a1.translate(instanceList=('cap-2-1', ), vector=(-55.0, 0.0,
↪  100.0)) #change last coordinate from vector according to your
↪  module's length
a1 = mdb.models['Model-%d' %(o+1)].rootAssembly
a1.InstanceFromBooleanMerge(name='Merged',
↪  instances=(a1.instances['Body-1'],
    a1.instances['cap-1-1'], a1.instances['cap-2-1'], ),
    keepIntersections=ON, originalInstances=SUPPRESS,
    ↪  domain=GEOMETRY)
```

```
    p1 = mdb.models['Model-%d' %(o+1)].parts['Merged']


############# CREATE SECTION   #############
    mdb.models['Model-%d'
    ↪  %(o+1)].HomogeneousSolidSection(name='silicone',
       material='silicone', thickness=None)
    p = mdb.models['Model-%d' %(o+1)].parts['Merged']
    c = p.cells
    cells = c.getSequenceFromMask(mask=('[#7 ]', ), )
    region = p.Set(cells=cells, name='Set-1')
    p = mdb.models['Model-%d' %(o+1)].parts['Merged']


############# ASSIGN SECTION  #############
    p.SectionAssignment(region=region, sectionName='silicone',
    ↪  offset=0.0,
       offsetType=MIDDLE_SURFACE, offsetField='',
       thicknessAssignment=FROM_SECTION)
    a = mdb.models['Model-%d' %(o+1)].rootAssembly
    a.regenerate()


############# CREATE STEPS  #############
    mdb.models['Model-%d' %(o+1)].StaticStep(name='gravity',
    ↪  previous='Initial',
       initialInc=0.1, nlgeom=ON)
    mdb.models['Model-%d' %(o+1)].Gravity(name='gravity',
    ↪  createStepName='gravity',
       comp3=9810.0, distributionType=UNIFORM, field='')
    a = mdb.models['Model-%d' %(o+1)].rootAssembly
    f1 = a.instances['Merged-1'].faces
    faces1 = f1.getSequenceFromMask(mask=('[#10 ]', ), )
    region = a.Set(faces=faces1, name='base')
    mdb.models['Model-%d' %(o+1)].EncastreBC(name='BC-1',
    ↪  createStepName='gravity',
       region=region, localCsys=None)
    mdb.models['Model-%d' %(o+1)].StaticStep(name='pressure_chambers',
    ↪  previous='gravity',
       initialInc=0.1)
    a = mdb.models['Model-%d' %(o+1)].rootAssembly
    s1 = a.instances['Merged-1'].faces
    side1Faces1 = s1.getSequenceFromMask(mask=('[#3c00041 ]', ), )
    region = a.Surface(side1Faces=side1Faces1, name='one_chamber')
    mdb.models['Model-%d' %(o+1)].Pressure(name='Load-2',
```

```
        createStepName='pressure_chambers', region=region,
        distributionType=UNIFORM, field='', magnitude=0.05,
        ↪  amplitude=UNSET) #insert your desired pressure in MPa
    mdb.models['Model-%d'
    ↪  %(o+1)].loads['Load-2'].setValues(magnitude=0.05)  #insert
    ↪  your desired pressure in MPa


############# CREATE MESH  #############
    p = mdb.models['Model-%d' %(o+1)].parts['Merged']
    c = p.cells
    pickedRegions = c.getSequenceFromMask(mask=('[#7 ]', ), )
    p.setMeshControls(regions=pickedRegions, elemShape=TET,
    ↪  technique=FREE)
    elemType1 = mesh.ElemType(elemCode=C3D20R)
    elemType2 = mesh.ElemType(elemCode=C3D15)
    elemType3 = mesh.ElemType(elemCode=C3D10)
    p = mdb.models['Model-%d' %(o+1)].parts['Merged']
    c = p.cells
    cells = c.getSequenceFromMask(mask=('[#7 ]', ), )
    pickedRegions =(cells, )
    p.setElementType(regions=pickedRegions, elemTypes=(elemType1,
    ↪  elemType2,
        elemType3))
    p = mdb.models['Model-%d' %(o+1)].parts['Merged']
    p.seedPart(size=2, deviationFactor=0.1, minSizeFactor=0.1) #insert
    ↪  your desired mesh seed size
    p = mdb.models['Model-%d' %(o+1)].parts['Merged']
    p.generateMesh()
    elemType1 = mesh.ElemType(elemCode=C3D20R, elemLibrary=STANDARD)
    elemType2 = mesh.ElemType(elemCode=C3D15, elemLibrary=STANDARD)
    elemType3 = mesh.ElemType(elemCode=C3D10H, elemLibrary=STANDARD)
    p = mdb.models['Model-%d' %(o+1)].parts['Merged']
    c = p.cells
    cells = c.getSequenceFromMask(mask=('[#7 ]', ), )
    pickedRegions =(cells, )
    p.setElementType(regions=pickedRegions, elemTypes=(elemType1,
    ↪  elemType2,
        elemType3))
    a = mdb.models['Model-%d' %(o+1)].rootAssembly
    a.regenerate()


############# CREATE JOBS  #############
    mdb.Job(name='Job_for_Model_%d' %(o+1), model='Model-%d' %(o+1),
    ↪  description='',
```

```
        type=ANALYSIS, atTime=None, waitMinutes=0, waitHours=0,
         ↪  queue=None,
        memory=90, memoryUnits=PERCENTAGE, getMemoryFromAnalysis=True,
        explicitPrecision=SINGLE, nodalOutputPrecision=SINGLE,
         ↪  echoPrint=OFF,
        modelPrint=OFF, contactPrint=OFF, historyPrint=OFF,
         ↪  userSubroutine='',
        scratch='', resultsFormat=ODB, multiprocessingMode=DEFAULT,
         ↪  numCpus=8, #insert your number of CPUs
        numDomains=8, numGPUs=0)
    mdb.jobs['Job_for_Model_%d'
     ↪  %(o+1)].submit(consistencyChecking=OFF)
    mdb.jobs['Job_for_Model_%d' %(o+1)].waitForCompletion()
    session.mdbData.summary()
    a = mdb.models['Model-%d' %(o+1)].rootAssembly
```
